# The PRO-RCC study: a long-term PROspective Renal Cell Carcinoma cohort in the Netherlands, providing an infrastructure for ‘Trial within Cohorts’ study designs

**DOI:** 10.1186/s12885-023-11094-9

**Published:** 2023-07-11

**Authors:** Hilin Yildirim, Christiaan V Widdershoven, Maureen JB Aarts, Axel Bex, Haiko J Bloemendal, Deirdre M Bochove-Overgaauw, Paul Hamberg, Karin H Herbschleb, Tom van der Hulle, Brunolf W Lagerveld, Martijn GH van Oijen, Sjoukje F Oosting, Johannes V van Thienen, Astrid AM van der Veldt, Hans M Westgeest, Evelijn E Zeijdner, Katja KH Aben, Corina van den Hurk, Patricia J Zondervan, Adriaan D Bins

**Affiliations:** 1 Department of Research and Development, Netherlands Comprehensive Cancer Organisation, Utrecht, The Netherlands; 2 Department of Medical Oncology, Cancer Center Amsterdam, Amsterdam UMC location University of Amsterdam, 4F De Boelelaan 1117, Amsterdam, 1081 HV The Netherlands; 3 Department of Urology, Amsterdam UMC location University of Amsterdam, Amsterdam, The Netherlands; 4 Department of Medical Oncology, GROW-School for Oncology and Development Biology, Maastricht University Medical Centre+, Maastricht, the Netherlands; 5 Department of Urology, The Netherlands Cancer Institute, Antoni van Leeuwenhoek Hospital, Amsterdam, The Netherlands; 6 The Royal Free London NHS Foundation Trust, London, UK; 7 UCL Division of Surgery and Interventional Science, London, UK; 8 Department of Oncology, Radboud University Medical Centre, Nijmegen, The Netherlands; 9 Department of Urology, Gelre Hospitals, Apeldoorn/Zutphen, The Netherlands; 10 Department of Internal Medicine, Franciscus Gasthuis & Vlietland, Rotterdam/Schiedam, the Netherlands; 11 Department of Internal Medicine, St. Antonius Ziekenhuis, Nieuwegein, The Netherlands; 12 Department of Medical Oncology, LUMC, Leiden, the Netherlands; 13 Department of Urology, OLVG, Amsterdam, The Netherlands; 14 Department of Medical Oncology, University Medical Center Groningen, University of Groningen, Groningen, The Netherlands; 15 Department of Medical Oncology, Netherlands Cancer Institute, Antoni van Leeuwenhoek hospital, Amsterdam, The Netherlands; 16 Department of Medical Oncology, Department of Radiology & Nuclear Medicine, Erasmus Medical Center-Cancer Institute, Rotterdam, the Netherlands; 17 Department of Internal Medicine, Amphia Hospital, Breda, the Netherlands; 18 Dutch Oncology Research Platform, Utrecht, The Netherlands; 19 Department for Health Evidence, Radboud University Medical Centre, Nijmegen, The Netherlands

**Keywords:** Kidney cancer, Renal cell carcinoma, Urological cancer

## Abstract

**Background:**

Ongoing research in the field of both localized, locally advanced and metastatic renal cell carcinoma has resulted in the availability of multiple treatment options. Hence, many questions are still unanswered and await further research. A nationwide collaborative registry allows to collect corresponding data. For this purpose, the Dutch PROspective Renal Cell Carcinoma cohort (PRO-RCC) has been founded, for the prospective collection of long-term clinical data, patient reported outcome measures (PROMs) and patient reported experience measures (PREMs).

**Methods:**

PRO-RCC is designed as a multicenter cohort for all Dutch patients with renal cell carcinoma (RCC). Recruitment will start in the Netherlands in 2023. Importantly, participants may also consent to participation in a ‘Trial within cohorts’ studies (TwiCs). The TwiCs design provides a method to perform (randomized) interventional studies within the registry. The clinical data collection is embedded in the Netherlands Cancer Registry (NCR). Next to the standardly available data on RCC, additional clinical data will be collected. PROMS entail Health-Related Quality of Life (HRQoL), symptom monitoring with optional ecological momentary assessment (EMA) of pain and fatigue, and optional return to work- and/or nutrition questionnaires. PREMS entail satisfaction with care. Both PROMS and PREMS are collected through the PROFILES registry and are accessible for the patient and the treating physician.

**Trial registration:**

Ethical board approval has been obtained (2021_218) and the study has been registered at ClinicalTrials.gov (NCT05326620).

**Discussion:**

PRO-RCC is a nationwide long-term cohort for the collection of real-world clinical data, PROMS and PREMS. By facilitating an infrastructure for the collection of prospective data on RCC, PRO-RCC will contribute to observational research in a real-world study population and prove effectiveness in daily clinical practice. The infrastructure of this cohort also enables that interventional studies can be conducted with the TwiCs design, without the disadvantages of classic RCTs such as slow patient accrual and risk of dropping out after randomization.

## Background

Worldwide, approximately 400 000 people are diagnosed with renal cell carcinoma (RCC) every year. This makes RCC the seventh most common form of neoplasm in the developed world, associated with more than 140 000 annual deaths [1]. In the Netherlands more than 2600 patients are diagnosed with RCC every year [2]. Over the past decades there is an increasing incidence of RCC in high-income countries, mostly due to the incidental detection of renal masses with abdominal imaging. As a result, renal masses are increasingly diagnosed at an early stage [3].

Treatment modalities for RCC have significantly developed over the last decades. However, many questions remain unanswered, such as the role of cytoreductive nephrectomy, the role of peri-operative treatment and the optimal sequence of systemic therapies [4]. Furthermore, the best strategy for follow-up should be evaluated.

For localized RCC, nephron-sparing (robot-assisted) partial nephrectomy has become common practice. In addition, ablative techniques and active surveillance for small renal masses (SRM) have entered daily practice [5, 6]. Ablative techniques have shown to be a minimally invasive and safe treatment option for SRM in terms of complications, adverse events and early recurrence rates. However, oncological outcomes remain unclear[7], and it’s long-term impact on health-related quality of life (HRQoL). Furthermore, active surveillance has proven to be a safe management for SRM, especially for older patients with comorbidities [8]. With several treatment options for localized RCC, more insight is warranted into finding the optimal care for the individual patient.

It is known that approximately one-third of the patients with RCC present with metastatic disease at diagnosis [1]. Immune therapy and targeted treatments have dramatically changed the treatment landscape for patients with metastatic RCC (mRCC) [9]. Interferon alpha and interleukin-2 were the mainstay of treatment and have been largely replaced in the last decades by vascular endothelial growth factor (VEGF) targeted therapies, mamalian target of rapamycin (mTOR) inhibitors and immune checkpoint inhibitors (ICI). Data from randomized controlled trials (RCTs) have shown higher response rates and improved clinical outcomes for these novel therapies [10–12].

Ideally, all new treatments should be compared to the standard of care in RCTs to determine efficacy. However, concurrent development of multiple new systemic therapies has resulted in a situation where most first line options have not been compared head-to-head. Also, data derived from RCTs are not completely generalizable to the real world practice, as it is known that a highly selected population is participating in clinical trials [9]. Only 5–15% of the patient population is participating in clinical trials, impeding representativeness [13, 14]. As example, it is known that the median age of cancer trial participants is on average seven years younger compared to the general cancer patient population [15, 16]. Thus, although data derived from RCTs can prove efficacy in a selective study population, such data do not prove effectiveness in daily clinical practice. Therefore, it is important to validate data from RCTs in observational research with real world data.

An alternative to classic RCTs are interventional studies with the Trial within Cohorts (TwiCs) design, also known as ‘cohort multiple randomized controlled trials’ (cmRCT) [17, 18]. Cohort participants will be selected based on their eligibility and randomized to a control or intervention arm at one moment in time. The TwiCs design eliminates some issues that are experienced in RCTs, such as slow patient accrual and risk of dropping out due to disappointment after randomization.

In summary, the PROspective Renal Cell Carcinoma cohort (PRO-RCC) is an initiative to construct a nationwide long-term cohort of RCC patients in the Netherlands, enabling collection of long-term clinical data, patient reported outcome measures (PROMs) and patient reported experience measures (PREMs) to facilitate observational research and fill in remaining gaps in the field of RCC to improve HRQol and quality of care of all patients with RCC. Furthermore, interventional research can be conducted using the TwiCs design[17], which is embedded in the PRO-RCC infrastructure.

## Methods and design

### Inclusion of patients and informed consent

Our observational cohort is designed for continuous inclusion and longitudinal follow-up of newly diagnosed patients with RCC in the Netherlands (localized, locally advanced or with synchronous metastases) and also for patients with metachronous metastases. All Dutch inhabitants from the age of 18 years with histologically proven or high clinical suspicion of (m)RCC are eligible for inclusion. Patients should be able to understand (written) Dutch language for participation. Written informed consent is mandatory for participation in the observational cohort. Study information is provided to each eligible patient by the treating physician or research nurse after initial diagnosis, but before the start of treatment during regular out-patient visits of the patient in the participating hospital. Patients will receive sufficient time to consider their participation.

**Fig. 1:**
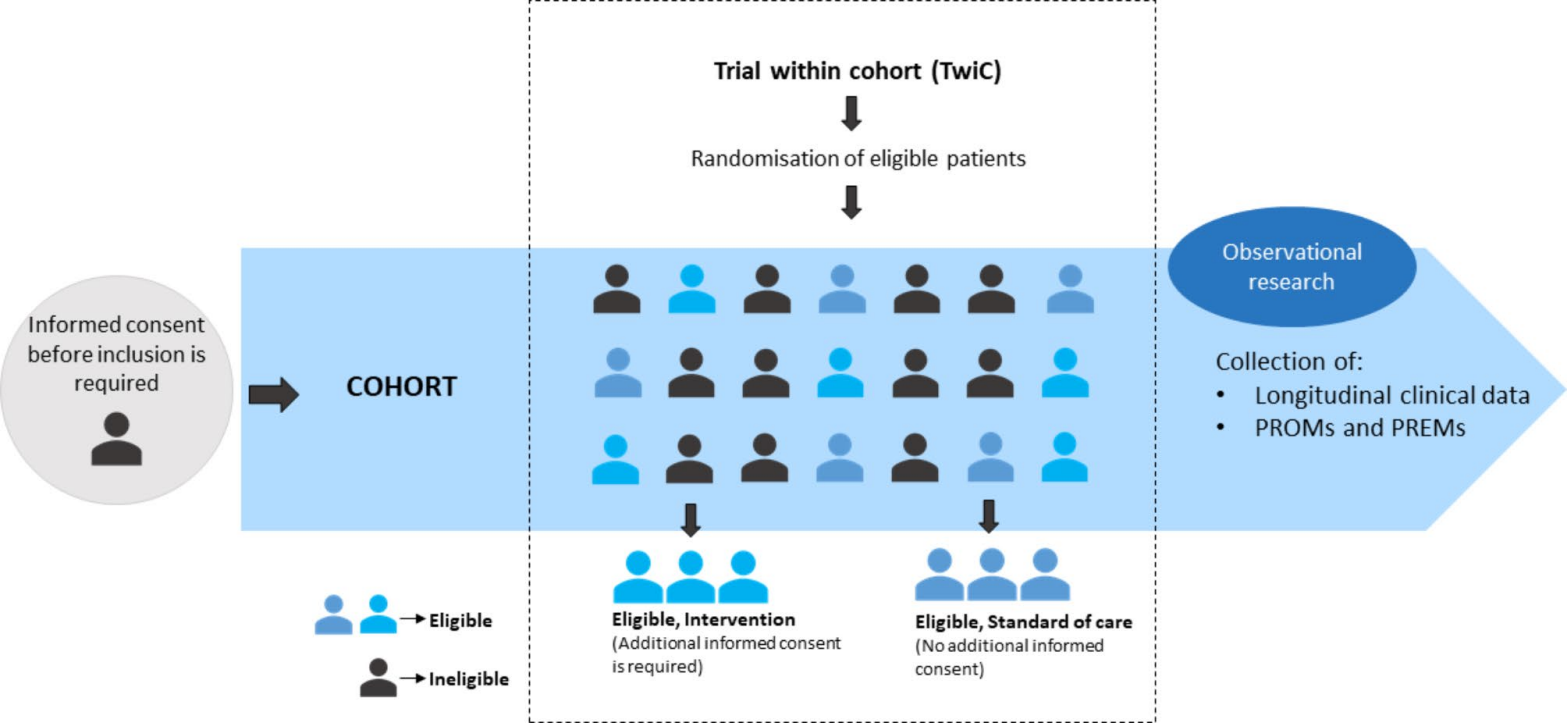
The ‘Trial within Cohorts’ design (TwiCs). After ethical approval for an interventional TwiCs study, eligible patients are selected from the cohort. Only patients who have given informed consent for potential participation in TwiCs upon entry in the PRO-RCC cohort are eligible for selection. After selection, eligible patients are randomized to the control or the intervention arm. Before inclusion in the intervention arm, separate informed consent has to be obtained from each patient. Patients in the control arm receive standard of care similar to the rest of the cohort and do not receive additional information. These patients have consented not to be notified of randomization in a TwiCs control arm, as part of their consent to TwiCs participation upon entry in the PRO-RCC cohort

Informed consent is given for the collection of PROMs, PREMs, (clinical) data sharing and data linking with the Netherlands Cancer Registry (NCR). Secondly, patients can opt in for potential participation in TwiCs. TwiCs can be conducted within the infrastructure of the cohort. Subjects who opt in for potential TwiCs participation, consent not to receive additional information if they are randomized to the control arm of a TwiCs study within the cohort. Only patients who are randomized for the interventional arm of a TwiCs will be informed about the study and receive additional study information. Before enrollment in the interventional arm additional informed consent for the specific TwiCs study is mandatory (Fig. 1). Medical ethical approval of an institutional review board is required at initiation of each TwiCs, as is the case with any regular RCT. There is no limitation for participation of patients in randomized trials unrelated to PRO-RCC and patients can participate in different TwiCs at the same time, unless explicitly stated otherwise in a specific TwiCs protocol in which the patient is participating.

### Proceedings

Recruitment will start in the Netherlands in 2023. Nationwide expansion of participating hospitals is planned for subsequent years. Over 30 hospitals (community and academic) throughout the Netherlands have expressed their intention to participate in the PRO-RCC cohort. It is our aim to include approximately 70% of all newly diagnosed RCC cases in the Netherlands in PRO-RCC (approximately 1900 per year in case all hospitals in the Netherlands participate in PRO-RCC). No end date or recruitment target has been specified.

Before the start of prospective inclusion, a pilot study was performed. Extensive clinical data, largely according to the item list as defined for PRO-RCC, of approximately 150 patients were retrospectively collected from 2019 to 2020 for first evaluation. Based on the findings of this pilot, the final item list has been established. As the number of prospectively included patients in PRO-RCC will gradually increase over the years, we performed an additional data collection of all patients diagnosed with mRCC in 2018, 2019 and 2020 (N ~ 1500). This data collection will provide the opportunity to study relevant research questions on a short term.

### Clinical data collection

Clinical data collection is embedded in the NCR which is maintained by the Netherlands Comprehensive Cancer Organization (IKNL) [19]. The NCR has nationwide coverage since 1989. Well-trained data managers collect data of all patients diagnosed with cancer in the Netherlands. These data include patient- and tumor characteristics (comorbidities, morphology, RENAL score, PADUA score), disease stage (clinical and pathological TNM stage, WHO/ISUP grade) and treatment (type of surgery, surgical margins, type of systemic therapy). Vital status is recorded based on annual linkage with the Municipal Personal Records Database which holds information on vital status and emigration of all Dutch inhabitants.

Additional PRO-RCC specific clinical items, such as laboratory tests, complications/toxicity, and specific details consisting systemic therapy, are collected from the medical files by the data managers.

In case of localized RCC, the following laboratory results are recorded: hemoglobin, creatinine and eGFR at diagnosis and at 6 months after local treatment. In case of mRCC hemoglobin, creatinine, eGFR, thrombocytes, neutrophils, calcium, albumin and LDH measurements are retrieved from the medical file at diagnosis, and before the start of each line of a systemic treatment.

Furthermore, 30 day-complications and the Clavien Dindo grade are registered for local treatments. The additional collection of items on systemic treatment consists of specific details concerning systemic therapy (e.g. dose, number of cycles, modifications, complications/toxicity, use of immunosuppressive drugs), clinical response to the systemic therapy and unexpected emergency room visits and/or hospital admission.

Clinical data collection will start for all patients 7 months after diagnosis. Thereafter, for patients with mRCC additional data will be collected one year after the first registration at 7 months, and a last time shortly after the patients has been deceased. For localized RCC the exact data collections time points have yet to be determined.

### Collection of PROMs and PREMs

**Fig. 2:**
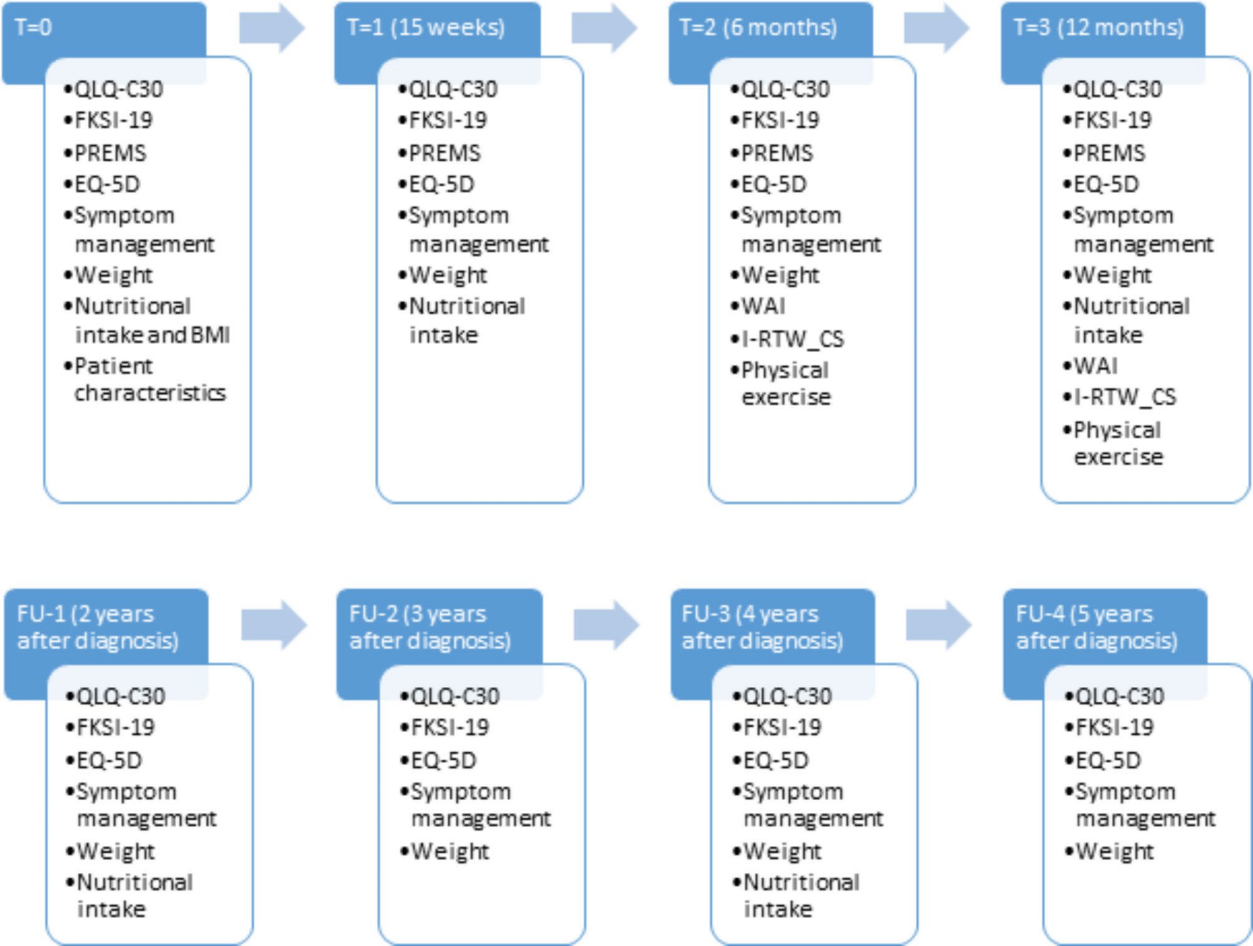
Overview of the collected patient reported oucome- and expierence measures over time. T = Time, FU = Follow-up

Patients participating in PRO-RCC will receive online PROMs and PREMs questionnaires. These questionnaires are collected with the Patient Reported Outcomes Following Initial treatment and Long term Evaluation of Survivorship (PROFILES) registry[20], which can be linked to the clinical data of the NCR. Information on comorbidity, marital status, educational level, and employment status will be registered in the baseline questionnaire. Furthermore, questionnaires on HRQoL, using the Dutch validated EORTC Quality of Life Questionnaire (QLQ-C30)[21] and Dutch version of the EuroQOL groups health status measure EQ-5D-5 L[22], will be collected at diagnosis, 15 weeks, 6 months, one year, and thereafter yearly until five years of follow-up or death (Fig. 2).

### Symptom monitoring

Patient-reported symptoms will be collected online through the newly developed ‘SYMPRO 2.0’ application (mobile website), which is incorporated in PROFILES[20, 23]. The SYMPRO 2.0 approach is described in detail elsewhere[24]. SYMPRO 2.0 allows the patient, treating physician and nurse to monitor symptoms. In the first year following diagnosis patients are requested to fill in a RCC specific symptom list, monthly in the first year, or more frequently if so desired by the participant with a maximum of once daily. The RCC specific symptom list will be collected yearly up to five years after the first year (Fig. 2). If patients receive any systemic therapy, then a treatment-specific symptom list, instead of the RCC specific symptom list, will be collected weekly up to one year. This symptom list is retrieved from the side-effects application ‘BijwerkingenBijKanker.nl’[25].

All symptoms are based on the patient-reported outcomes version of the common terminology criteria for adverse events (PRO-CTCAE) [26]. If necessary items were not available in the PRO-CTCAE, they were formulated in the PRO-CTCAE code by researchers and health care professionals and tested by health care professionals and laymen. The RCC-specific symptoms are based on the National Comprehensive Cancer Network/Functional Assessment of Cancer Therapy – Kidney Symptom Index 19 (FKSI-19)[27] and recoded into PRO-CTCAE items.

After each assessment by the patient, an overview of symptoms over time is available, which is also accessible online for the treating physicians and oncology nurses. Furthermore, alerts are forwarded to the patient if a selected symptom exceeds a particular threshold (such as the combination of diarrhea and vomiting for more than one day) or according to the composite grading algorithm that has been developed for the PRO-CTCAE[26]. If preferred in a participating hospital, alerts can also be forwarded by e-mail to the oncology nurse. Importantly, patients are instructed not to rely on this email system and to contact their physician in case of an alert and to follow the regular instructions provided at the treatment initiation.

### Collection of additional questionnaires regarding nutrition, fatigue and pain and return to work

Optionally, questionnaires of nutritional intake, fatigue, pain and return-to-work are assessed if the subject has consented to these in the PROFILES application. Nutrition will be monitored by registering all foods and drinks during two weekdays and one weekend day, using the ‘Eetmeter’ from the Netherlands Nutrition Centre [Dutch: Voedingscentrum][28]. After registration, results of the ‘Eetmeter’ are shared in the secure PROFILES[20] environment. It is requested to fill in the intake at diagnosis, after 15 weeks, and after one and three years (Fig. 2). On these moments waist and hip circumference will be measured using a flexible measuring tape that is provided to the patient. The waist circumference and waist-to-hip ratio gives a global indication of the intra-abdominal fat mass [29]. Decreased intake and cachexia can occur during systemic treatment of mRCC, in particular with targeted therapies. In order to interpret the results with a more complete overview of the lifestyle, the ‘SQUASH’ questionnaire on physical exercise is added[30].

If moderate or severe fatigue or pain has repeatedly been reported in the SYMPRO 2.0 app for a period of two weeks, then optionally patients can keep record of a detailed fatigue or pain diary using Ecological Momentary Assessment (EMA) with the Ethica application [31, 32]. The results of the questionnaires will be summarized in a report, that can be shared with the treating physician or nurse. Based on these data it will be possible to evaluate whether early detection of these symptoms can prevent further deterioration and maybe long-term complaints.

Questionnaires on return-to-work are optional if (1) there is an employment contract at diagnosis, and (2) if own or adjusted work activities have been performed in the 4 weeks preceding the diagnosis. If both requirements are met, patient can opt in for questionnaires on work at 6 months and 1 year after diagnosis. It is known that the impact of cancer on work can be significant[33]. The ‘Work Ability Index’ (WAI) and the ‘Successful Return-To-Work Questionnaire for Cancer Survivors’ (I-RTW_CS) will be used[33, 34].

### PREMS

PREMs will be collected at diagnosis, 15 weeks, 6 months and one year, consisting of several questions regarding satisfaction with care. This questionnaire has been developed by the Dutch Federation of Cancer Patient Organizations [Dutch: Nederlandse Federatie van Kankerpatienten organisaties] (NFK)[35] in collaboration with the Dutch patient association for bladder or kidney cancer [Dutch: Leven met blaas- of nierkanker][36].

### Governance

PRO-RCC is registered as foundation and is governed by committees consisting of medical oncologists, urologists and epidemiologists. A separate scientific advisory committee reviews new research proposals based on scientific value.

### Patient and public involvement

The Dutch patient association for bladder or kidney cancer (Dutch: Leven met Blaas- of Nierkanker) were consulted in setup of the study. They will continue on providing input on scientific priorities, as they are a part of the PRO-RCC scientific advisory committee. Furthermore, they facilitate communication of study findings to patients.

### Safety

Participants can withdraw from the project or related studies at any time and for any reason without any consequences. The non-interventional nature of this registry precludes the occurrence of adverse events as a result of participation. However, as mentioned above, there will be monitoring of symptoms from received treatments during this study through PROFILES. This will not be used as replacement for instructions from the treating physician and nurse. The symptom monitoring can be used as add-on in the outpatient clinic. Patients will receive instructions concerning how and when to contact their treating physician with any alert. Furthermore, data on symptoms and medication agent(s) will be shared with pharmacovigilance centre Lareb[37]. Lareb will monitor the data for outliers and discrepancies in prevalence of symptoms compared to the summaries of product characteristics of the particular agents.

## Discussion

The aim of PRO-RCC is to construct a nationwide cohort for patients with (m)RCC to collect real-world clinical data, PROMs and PREMs to facilitate observational research and provide a platform for interventional studies with the TwiCs design. Furthermore, PRO-RCC encourages data sharing and collaborations with external groups. Similar initiatives in the Netherlands have already proven its efficacy in the Dutch ‘Prospective Bladder Cancer Infrastructure’ (ProBCI) [38, 39] and in the Dutch ‘Prospective Nationwide Colorectal cancer Cohort’ (PLCRC) [40, 41].

PRO-RCC aims to achieve high recruitment rates within the cohort, allowing to conduct sufficient data analyses and providing an infrastructure for interventional studies. Such interventional studies can be conducted within PRO-RCC using the TwiCs design. The main advantage of TwiCs is improved recruitment rates, as eligible patients can be selected from the cohort database. Additional informed consent is only necessary from patients randomized for intervention. Therefore, the TwiCs design eliminates the barrier for patients to consent to randomization with the risk of not being offered the preferred treatment [42]. Furthermore, the database provides an adequate representative sample of the control group with less selectiveness, as these patients cannot withdraw due to e.g. disappointment. The approach enables more direct and indirect comparisons, as all treatments have the same “treatment as usual” comparator and use the same core outcomes. The TwiCs design is only suited for interventional trials that compare the experimental arm to ‘standard of care’ and for research questions with outcomes that are easily measured and collected within the entire cohort. Also, the feasibility of conducting a TwiCs study hinges on the ability to identify eligible patients for the study in the cohort. Consequently, if the inclusion criteria cannot be established within the cohort, the execution of a TwiCs study becomes unfeasible. For instance, the identification and selection of patients with synchronous metastatic RCC and/or metachronous metastatic RCC are achievable within the cohort.

Furthermore, the treatment that is offered should have high acceptability. It should be noted that not all research questions can be addressed in a TwiCs design, such as closed trial designs with masking or a placebo arm. In addition, TwiCs with treatments not desired by patients are less suitable as there could be difficulties with recruitment in the interventional arm [17].

The clinical data collection of all patients diagnosed with mRCC in the period 2018–2020 through the NCR will enable analyses in a real world setting in short time. Starting in 2023 clinical data of newly diagnosed patients will be collected. These real-world clinical data will contribute to the knowledge of effectiveness of treatments in daily clinical practice. Furthermore, the cohort will enable subgroup analyses, as specific treatments are not always analyzed in subgroups, such as in non-clear-cell RCC or in older patients, as both groups are underrepresented in clinical trials.

In addition, the cohort will provide sufficient data on HRQOL and health care costs of different (novel) treatments, which are important considerations in (shared) decision making.

Altogether, the PRO-RCC cohort will provide a nationwide infrastructure for observational and interventional research, contributing to the evidence of clinical practice and creating opportunities for improvement of quality of care and quality of life of patients with RCC.

## Data Availability

The datasets generated and/or analyzed during the current study are not publicly available due to privacy but are available from the corresponding author on reasonable request. The original study protocol of this study is accessible upon request.

## Declarations

### Dissemination policy

All scientific output and developments concerning PRO-RCC will be publicly available through the project website (www.pro-rcc.nl). The Dutch PRO-RCC newsletter is open for registration to all patients, clinicians, and researchers who express interest in subscribing.

## List of abbreviations

*EMA*: Ecological Momentary Assessment
*HRQoL*: Health-Related Quality of Life
*IKNL*: Netherlands Comprehensive Cancer Organization
*mRCC*: Metastatic Renal Cell Carcinoma
*NCR*: Netherlands Cancer Registry
*NFK*: Dutch Federation of Cancer Patient Organizations
*PRO-RCC*: PROspective Renal Cell Carcinoma cohort
*PROMs*: Patient Reported Outcome Measures
*PREMs*: Patient Reported Experience Measures
*PRO-CTCAE*: Patient Reported Outcomes version of the common terminology criteria for adverse events
*PRO-BCI*: Prospective Bladder Cancer Infrastructure
*PLCRC*: Prospective Nationwide Colorectal cancer Cohort
*PROFILES*: Patient Reported Outcomes Following Initial treatment and Long term Evaluation of Survivorship
*RCC*: Renal Cell Carcinoma
*RCTs*: Randomized Controlled Trials
*SRM*: Small Renal Masses
*TwiCs*: Trial within Cohorts
